# Gestational diabetes increases preterm membrane rupture risk through elevated hydroxydesmethylpiperine sulfate levels

**DOI:** 10.3389/fendo.2025.1631638

**Published:** 2025-09-02

**Authors:** Ying Li, Rong Lin, Tianshu Zhang, Tianyu Shi, Xingwang Ding, Jinqi Ma

**Affiliations:** ^1^ Department of Obstetrics and Gynecology, The Affiliated Wuxi People’s Hospital of Nanjing Medical University, Wuxi People’s Hospital, Wuxi Medical Center, Nanjing Medical University, Wuxi, Jiangsu, China; ^2^ Center of Clinical Research, The Affiliated Wuxi People’s Hospital of Nanjing Medical University, Wuxi People’s Hospital, Nanjing Medical University, Wuxi, Jiangsu, China

**Keywords:** gestational diabetes mellitus (GDM), premature rupture of membranes (PROM), metabolism, biomarker, hydroxydesmethylpiperine sulfate

## Abstract

**Objectives:**

Gestational diabetes mellitus (GDM) is a metabolic disorder that increases the risk of premature rupture of membranes (PROM). The link between GDM-associated metabolic dysregulation and PROM remains unclear. This study investigates the underlying metabolic mechanisms to identify potential therapeutic targets for improving pregnancy outcomes.

**Methods:**

The study involved 354 mothers from the GDM Mother and Child Study (GMCS) at Wuxi People’s Hospital, Nanjing Medical University. A regression model assessed the GDM-PROM relationship. Third-trimester serum metabolites were analyzed using ultra-high-performance liquid chromatography and high-resolution mass spectrometry.

**Results:**

A higher percentage of women with PROM were diagnosed with GDM (15.7% vs. 32.3%, P=0.005). Although no significant differences were found in 1-h and 2-h glucose levels (P=0.216 and 0.129), fasting glucose was elevated in the PROM group (4.50 [0.43] vs. 4.73 [0.71], P=0.017). Both unadjusted and adjusted models confirmed GDM as a risk factor for PROM (OR: 2.548, 95% CI: 1.341–4.759; P=0.004). After adjusting for confounders, GDM remained positively associated with PROM risk (OR: 2.538, 95% CI: 1.223–5.224; P=0.012). Hydroxydesmethylpiperine sulfate levels were significantly elevated in all study groups.

**Conclusion:**

GDM elevates PROM risk by disrupting fetal membrane integrity through metabolic alterations. Elevated hydroxydesmethylpiperine sulfate levels in GDM-PROM cases indicate its potential as a PROM risk biomarker, underscoring the importance of early metabolic screening and glycemic control. Further research should explore this metabolite’s mechanisms and therapeutic potential.

## 1 Introduction

Gestational diabetes mellitus (GDM) represents a significant metabolic condition marked by the onset of glucose intolerance specifically during pregnancy. This condition increases the risk of several obstetric complications, notably premature rupture of membranes (PROM), which can lead to adverse outcomes for both mother and baby (1). PROM refers to the early breaking of the fetal membranes before the onset of labor, which can lead to significant perinatal complications. These include an increased risk of intrauterine infections, preterm birth, and other maternal and neonatal health issues (2, 3). Its higher prevalence in GDM pregnancies underscores the need to understand the underlying metabolic mechanisms.

GDM-related PROM is primarily driven by metabolic disturbances, including hyperglycemia, oxidative stress, and inflammation (4, 5). Chronic hyperglycemia weakens fetal membranes by disrupting collagen integrity and increasing matrix metalloproteinase (MMP) activity (6). It also promotes advanced glycation end products (AGEs) accumulation, compromising membrane strength (7). Insulin resistance and lipid peroxidation produce reactive oxygen species (ROS), intensifying oxidative stress. This damages cellular structures, disrupts functions, and promotes inflammation, contributing to complications like premature rupture of membranes (PROM) and preeclampsia in pregnancy (8).

GDM induces a pro-inflammatory state with elevated cytokines like IL-6 and TNF-α, which degrade membrane structure (9, 10). Additionally, metabolic dysregulation alters amniotic fluid composition, leading to polyhydramnios and heightened mechanical stress on membranes (11). Moreover, impaired glucose metabolism affects nutrient transport and cellular signaling in placental and fetal tissues, disrupting protein and lipid synthesis essential for membrane integrity (12). Dysregulated insulin signaling further imbalances extracellular matrix synthesis and degradation, worsening membrane fragility (13).

Understanding these metabolic mechanisms is key to early risk identification and targeted prevention. This study explores the association between gestational diabetes mellitus (GDM) and preterm rupture of membranes (PROM), with a focus on clinical interventions. It also provides recommendations for future research aimed at improving maternal and fetal health outcomes. By examining the underlying mechanisms and potential strategies for prevention and management, this work seeks to contribute to better healthcare practices and outcomes for affected pregnancies.

## 2 Materials and methods

### 2.1 Study design and population

**Figure 1:**
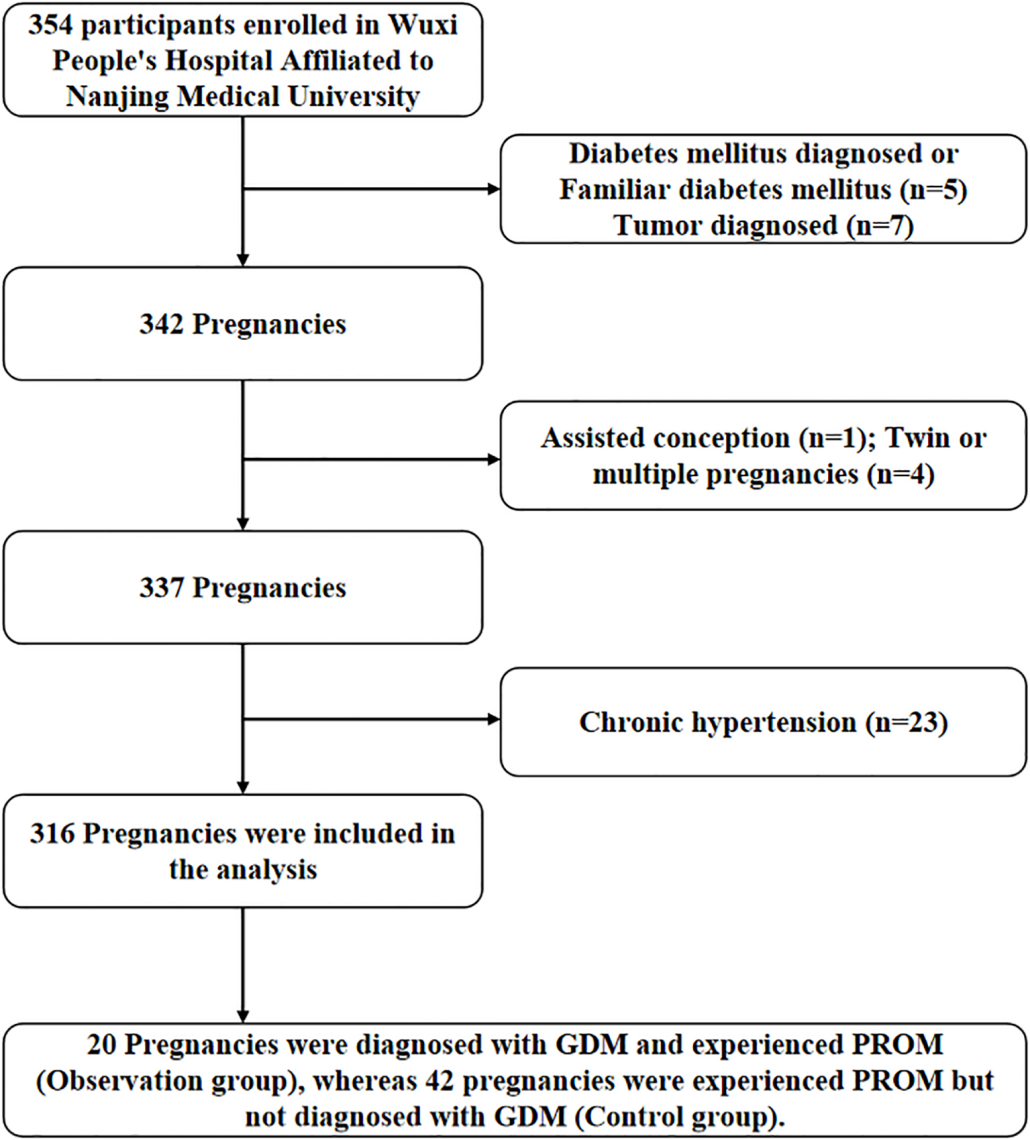
Flowchart of the participants included in the study.

Participants were recruited from GMCS at Wuxi People’s Hospital. The study was approved by the ethics committee of the hospital, ensuring adherence to ethical guidelines and the protection of participants’ rights throughout the research process (Ethical NO.: KY23120). All participants voluntarily provided written informed consent, confirming their comprehension of the study’s objectives, procedures, and potential risks. This ensured their informed and willing participation in the research. All participants provided informed consent and completed a 75-g oral glucose tolerance test (OGTT) during the 24th to 26th week of pregnancy. Gestational diabetes mellitus (GDM) was diagnosed based on the results of a 75-g OGTT conducted between the 24th and 26th week of pregnancy. The diagnostic criteria included: a fasting blood glucose level of 5.1 mmol/L or higher, a 1-hour post-glucose load level of 10.0 mmol/L or higher, or a 2-hour post-load level of 8.5 mmol/L or higher (14). This diagnostic approach ensured accurate identification of GDM, facilitating early intervention and management to reduce associated risks. Demographic and clinical characteristics, including maternal age, pre-pregnancy BMI, obstetric history, and familial diabetes background, were meticulously collected through structured questionnaires and electronic health records. This approach facilitated a detailed examination of relevant factors and their potential correlations with the study’s outcomes. Women with pre-existing diabetes, any metabolic disorders, recent use of antibiotics, a history of alcohol or drug abuse, or chronic health conditions that necessitated ongoing medication were excluded from the study to ensure the results were not influenced by these factors. Initially, a total of 354 pregnant women were recruited and enrolled in the study. After implementing stringent inclusion and exclusion criteria to ensure the relevance and reliability of the data, 316 women met the eligibility requirements and advanced to the follow-up phase. This careful selection process helped maintain the integrity of the study and ensured that the final participant pool was well-suited for detailed analysis and meaningful conclusions. Specific tolerance standards are shown in the figure below (**Figure 1**):

### 2.2 Clinical information collection and diagnosis of GDM

Clinical information was gathered through a detailed review of participants’ electronic medical records. The extracted data included maternal details (age, pre-pregnancy BMI, education level), pregnancy-related factors (gestational age, delivery mode, fetal sex, gestational hypertension, anemia, GDM), neonatal birth weight, and OGTT results (glucose levels at 0h, 1h, and 2h). To ensure data accuracy and completeness, trained researchers carefully extracted all relevant information.

### 2.3 Metabolomics detection

We matched the corresponding group samples according to the baseline characteristic propensity score of the population (1:1:1:1), and strategically selected 10 participants in each group, totaling 40 pregnant women in the third trimester. And metabolomics detection was conducted on it. Metabolite intensities were log-transformed and normalized using probabilistic quotient normalization (PQN) to account for dilution effects. Metabolites with >20% missing values were excluded; remaining missing values were imputed using k-nearest neighbor (k-NN) imputation. Data were adjusted using ComBat to correct for batch effects across sample runs. Principal component analysis (PCA) and orthogonal partial least squares discriminant analysis (OPLS-DA) were performed. Model validity was assessed via cross-validation and permutation testing. Differential metabolites were identified using adjusted p-values from univariate analysis (t-test or ANOVA) with false discovery rate (FDR) correction (Benjamini–Hochberg).

### 2.4 Statistical analysis

Descriptive statistics were applied to assess the baseline characteristics of the study participants, including key demographic factors such as age, body mass index (BMI), and gestational age, as well as other relevant clinical variables. This analysis provided a comprehensive overview of the participants’ profiles, facilitating a clearer understanding of the sample’s composition. Continuous variables were presented as mean ± standard deviation (mean ± SD), and differences between groups were assessed using independent t-tests. Categorical variables were expressed as percentages and analyzed using the Chi-squared test to evaluate statistical significance, allowing for comparison between the various groups within the study. These methods ensured a robust analysis of the data and facilitated the identification of meaningful differences across participant categories.

Logistic regression models were employed to investigate the association between PROM and GDM. The crude model (model-crude) did not adjust for any covariates. In model 1, potential confounders were included as covariates for adjustment. These covariates comprised maternal age, pre-BMI, gestational age, neonatal birth weight, maternal education level (categorized as college and below, university, and graduate school and above), fetal sex (boy vs. girl), mode of delivery (vaginal birth vs. cesarean section), gravidity (1 vs. ≥2), parity (0 vs. ≥1), gestational hypertension (yes vs. no), and anemia during pregnancy (yes vs. no). Missing covariate data were addressed using multiple imputation by chained equations (MICE). Odds ratios (OR) and their 95% confidence intervals (CI) were calculated. Additionally, subgroup analyses based on delivery mode (vaginal vs. cesarean), fetal sex (male vs. female), and pregnancy-related anemia (present vs. absent) were performed to further examine the relationship between GDM and PROM. All statistical analyses were conducted using R software (version 4.3.1), a widely recognized tool for robust data analysis. A P-value threshold of <0.05 was adopted to determine statistical significance, ensuring that observed differences between groups were unlikely to result from random variation. This criterion was rigorously applied to assess the strength of associations, validate the reliability of findings, and support the credibility of the study’s conclusions. Additionally, sensitivity analyses were performed to evaluate the consistency of results under varying assumptions, further enhancing the robustness of the statistical outcomes.

## 3 Results

### 3.1 Population characteristics

This study included 316 pregnant women, of whom 62 were diagnosed with GDM and 254 were healthy controls. The control group was used for comparison to assess the differences in various clinical and metabolic outcomes between the two groups, providing a comprehensive understanding of the impact of GDM on pregnancy. The results showed no significant differences in baseline characteristics between the two groups, including maternal age, BMI, gestational age, education level, gravidity, parity, infant birth weight, fetal sex, delivery mode, gestational hypertension, or anemia status. However, a significantly higher proportion of women with PROM had GDM, with 32.3% of the PROM group being GDM-positive compared to 15.7% in the non-PROM group (*P* = 0.005), suggesting a strong association between GDM and PROM. Further analysis revealed that fasting glucose levels were significantly higher in the PROM group (4.73 (0.71) mmol/L vs. 4.50 (0.43) mmol/L, *P* = 0.017). These findings suggest that GDM may raise the risk of PROM due to higher blood glucose levels, emphasizing the need for careful blood sugar monitoring in GDM patients throughout pregnancy to mitigate the risk of PROM.

### 3.2 Associations between GDM and PROM

Consistently demonstrated that gestational diabetes mellitus (GDM) is an independent risk factor for premature rupture of membranes (PROM); These findings underscore the significant link between elevated maternal blood glucose levels during pregnancy and a higher risk of PROM, emphasizing the importance of glycemic control in prenatal care to reduce adverse pregnancy outcomes (OR: 2.548, 95% CI: 1.341, 4.759; *P* = 0.004). After adjusting for covariates such as maternal age, pre-BMI, gestational age, infant birth weight, maternal education level, fetal sex, delivery mode, gravidity, parity, gestational hypertension, and anemia, GDM remained significantly associated with an increased risk of PROM. This persistent relationship underscores the independent contribution of GDM to the likelihood of PROM, even when accounting for other potential influencing factors (OR: 2.538, 95%CI: 1.223, 5.224; *P* = 0.012).

Furthermore, subgroup analysis revealed that pregnant women diagnosed with GDM and concurrently suffering from anemia exhibited a markedly higher risk of developing PROM. This heightened risk underscores the compounded impact of these two conditions, suggesting that the coexistence of GDM and anemia may exacerbate maternal vulnerability to adverse pregnancy outcomes. This finding underscores the compounded impact of both conditions on maternal and fetal health, emphasizing the need for careful monitoring and management of these co-occurring risk factors during pregnancy; Pregnant women with GDM who have caesarean section have a higher risk of premature rupture of membranes (**Supplementary Table 1**).

### 3.3 Associations between GDM and PROM with metabolites

**Figure 2:**
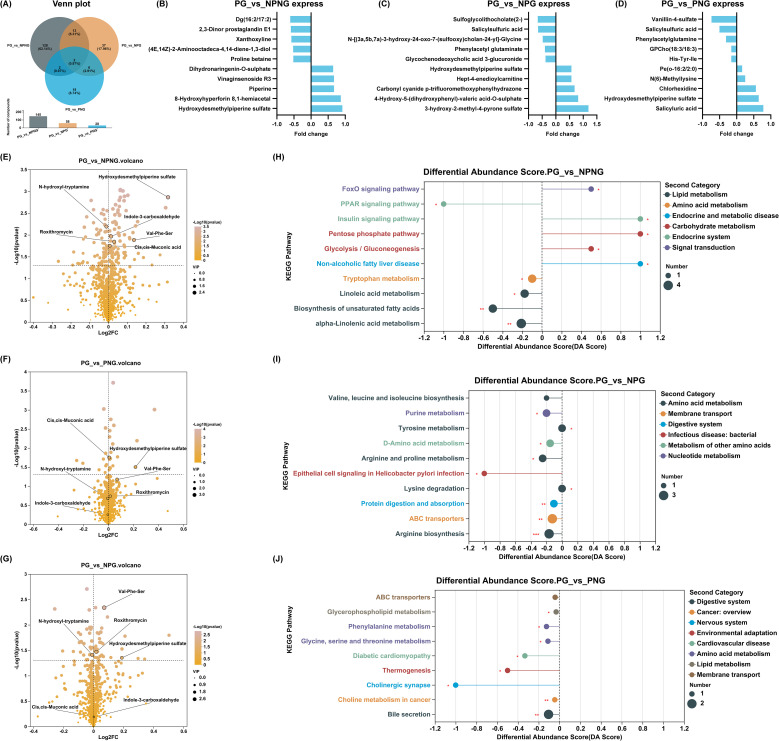
Associations between GDM and PROM with metabolites. **(A)** The Venn diagram of pairwise comparison. **(B–D)** Pairwise comparison of elevated and decreased TOP5 metabolites, **(B)** PG vs NPNG; **(C)** PG vs NPG; **(D)** PG vs PNG. **(E–G)** Volcanic map of differential metabolites in pairwise comparison, **(E)** PG vs NPNG; **(F)** PG vs PNG; **(G)** PG vs NPG. **(H–J)** Enrichment analysis of KEGG pathway in pairwise comparison, **(H)** PG vs NPNG; **(I)** PG vs NPG; **(J)** PG vs PNG. *P<0.05; **P<0.01; ***P<0.001.

To explore metabolic links, we further divided the population into four groups, namely NPNG (not experienced PROM and not diagnosed with GDM), PG (experienced PROM and diagnosed with GDM), PNG (experienced PROM but not diagnosed with GDM) and NPG (not experienced PROM but diagnosed with GDM) groups. Considering the notable link between GDM and PROM, we matched the corresponding group samples according to the baseline characteristic propensity score of the population (1:1:1:1), and strategically selected 10 participants in each group, totaling 40 pregnant women in the third trimester. The results showed no significant differences in baseline characteristics among the four groups, including maternal age, education level, gravidity, parity, fetal sex, delivery mode, gestational hypertension, or anemia status (**Supplementary Table 2**). These women underwent an in-depth metabolic analysis to investigate the potential involvement of specific metabolites in the development of GDM-induced PROM. In pound-for-pair comparative analysis, 145 (PG vs NPNG), 58 (PG vs NPG) and 28 (PG vs PNG) different metabolites were identified (**Figure 2A**). In the comparison between PG and NPNG group, the content of Dg (16:2/17:2), 2,3-Dinor prostaglandin E1, Xanthoxyline, (4E,14Z) -2-AminooctADECA-4, 14-Diene-1,3-diol and Proline betaine was significantly decreased; The content of Dihydronaringenin-O-sulfate, Vinaginsenoside R3, Piperine, 8-hydroxyhyperforin 8,1-hemiacetal and Hydroxydesmethylpiperine sulfate were significantly increased (**Figure 2B**). In the comparison of PG and NPG groups, the metabolite content of Sulfoglycolithocholate (2-), Salicylsulfuric acid, N-((3a,5b,7a) -3-hydroxy-24-OXo-7 -(sulfooxy)cholan-24-yl)-Glycine, Phenylacetyl glutaminate and Glycochenodeoxycholic acid 3-glucuronide was significantly decreased; Hydroxydesmethylpiperine sulfate, Hept - 4 - enedioylcarnitine, Carbonyl cyanide p - trifluoromethoxyphenylhydrazone, 4-Hydroxy-5-(dihydroxyphenyl) -Valeric acid-O-sulfate and 3-hydroxy-2-methyl-4-pyrone sulfate were significantly increased (**Figure 2C**). Metabolites contents of Vanillin-4-sulfate, Salicylsulfuric acid, Phenylacetylglutamine, GPCho (18:3/18:3) and His-Tyr-Ile were significantly decreased in PG group and PNG group; Pe (o-16:2/2:0), N (6)-Methyllysine, Chlorhexidine, Hydroxydesmethylpiperine sulfate and Salicyluric acid metabolites were significantly elevated (**Figure 2D**). Only Hydroxydesmethylpiperine sulfate metabolite content was significantly increased in the NPNG, PNG, NPG, and PG groups (**Figures 2E–G**). KEGG pathway enrichment analysis revealed that the differential metabolites were significantly enriched in several critical biological pathways. These included the FoxO signaling pathway, PPAR signaling pathway, and insulin signaling pathway, which are closely linked to metabolic regulation. Additionally, pathways associated with tryptophan metabolism, lysine degradation, and phenylalanine metabolism were also prominently represented (**Figures 2H–J**). These pathways may be tightly interconnected with both GDM and PROM, highlighting potential metabolic disruptions contributing to the development of PROM in GDM-affected pregnancies.

## 4 Discussion

Findings confirm a strong correlation between GDM and PROM, underscoring the role of metabolic dysregulation in membrane fragility. The increased incidence of PROM in GDM pregnancies highlights the necessity of early metabolic monitoring and intervention to mitigate adverse outcomes. Further analysis suggests that GDM may serve as an independent risk factor for PROM. Despite rigorous adjustment for a range of potential confounding factors, including maternal age, pre-BMI, gestational age, infant birth weight, maternal education level, fetal sex, delivery mode, gravidity, parity, gestational hypertension, and anemia, the association between GDM and membrane rupture remains robust and statistically significant. This persistent relationship underscores the independent contribution of GDM to the risk of membrane rupture, highlighting the necessity of implementing tailored screening protocols and personalized management strategies for pregnancies at elevated risk. Such measures are essential to mitigate adverse outcomes and ensure optimal maternal and fetal health. Subgroup analysis also reveals that gestational anemia contributes to an elevated risk of PROM, indicating that maternal nutritional status should be considered in risk assessments. This finding suggests a potential interplay between iron metabolism, oxygen transport, and membrane integrity, warranting further investigation (15, 16).

Additionally, metabolic profiling indicates that hydroxydesmethylpiperine sulfate levels are elevated in both GDM and PROM groups. This metabolite may be linked to alterations in gut microbiota, which are increasingly recognized as key regulators of maternal-fetal health (17, 18). Dysbiosis in the gut may lead to increased intestinal permeability and systemic inflammation, influencing metabolic pathways that impact membrane integrity (19–21). Meanwhile, vaginal microbiota imbalances may lead to an overgrowth of pathogenic bacteria, triggering localized inflammation and enzymatic degradation of membrane components (22–24). These insights highlight the complex interactions between metabolic pathways and microbial ecosystems in pregnancy.

Hydroxydesmethylpiperine sulfate may also directly affect placental and fetal membrane function by modulating metabolic and inflammatory signaling pathways (25, 26). Increased levels of this metabolite could disrupt placental transport mechanisms, altering nutrient and oxygen exchange essential for fetal development. Furthermore, its potential interaction with inflammatory mediators may enhance oxidative stress and weaken extracellular matrix stability within the fetal membranes (27, 28). This metabolic disruption may also impair trophoblast function, leading to abnormal hormone secretion and placental insufficiency, further contributing to PROM risk (6, 29, 30). Additionally, hydroxydesmethylpiperine sulfate may influence epithelial barrier integrity in both the placenta and fetal membranes, making them more susceptible to premature rupture (26).

The identification of hydroxydesmethylpiperine sulfate as a potential biomarker for GDM-related PROM suggests novel diagnostic and therapeutic possibilities. Targeting microbial dysbiosis through probiotics, prebiotics, or microbiome-modulating interventions could offer new avenues for preventing PROM in high-risk pregnancies. Additionally, metabolic interventions aiming to reduce oxidative stress and regulate inflammatory responses may play a critical role in membrane stabilization. Future research should focus on understanding the specific mechanisms by which microbial metabolism influences fetal membrane integrity in GDM patients. Additionally, it is crucial to explore potential therapeutic interventions that could restore a healthy microbial balance, improving outcomes for both mothers and infants.

This metabolite may also interfere with placental transport, impair trophoblast function, and influence extracellular matrix stability. A comprehensive approach incorporating glycemic control, nutritional optimization, and microbiota-associated signatures may help reduce the incidence of PROM in GDM pregnancies. To further validate and extend our findings, future studies will employ targeted mass spectrometry to precisely quantify hydroxydesmethylpiperine sulfate levels in a larger independent cohort, ensuring accurate measurement. Additionally, replication in separate populations with diverse backgrounds will be conducted to assess the robustness and generalizability of the observed association. Finally, the metabolite’s diagnostic or predictive potential will be rigorously evaluated through ROC curve analysis and machine learning-based classification models, providing a comprehensive assessment of its clinical utility. Further research is also needed to elucidate the mechanistic pathways and develop effective prevention strategies.

## 5 Conclusion

In summary, GDM significantly increases the risk of PROM through metabolic disruptions that compromise fetal membrane integrity. Elevated hydroxydesmethylpiperine sulfate levels in GDM and PROM patients suggest a potential link between metabolic dysregulation, microbial imbalances, and membrane fragility. These findings highlight the need for early metabolic screening, improved glycemic control, and microbiota-targeted potential interventions to reduce PROM risk. Future research should further explore the mechanistic roles of this metabolite and develop effective therapeutic strategies to enhance maternal-fetal health outcomes.

## Data Availability

The raw data supporting the conclusions of this article will be made available by the authors, without undue reservation.
